# The HIV-1 Vpu Viroporin Inhibitor BIT225 Does Not Affect Vpu-Mediated Tetherin Antagonism

**DOI:** 10.1371/journal.pone.0027660

**Published:** 2011-11-14

**Authors:** Björn D. Kuhl, Vicky Cheng, Daniel A. Donahue, Richard D. Sloan, Chen Liang, John Wilkinson, Mark A. Wainberg

**Affiliations:** 1 McGill University AIDS Center, Lady Davis Institute, Jewish General Hospital, Montréal, Quebec, Canada; 2 Department of Experimental Medicine, McGill University, Montréal, Canada; 3 Department of Microbiology and Immunology, McGill University, Montréal, Canada; 4 Biotron Limited, St Vincent's Centre for Applied Medical Research, Sydney, Australia; George Mason University, United States of America

## Abstract

Among its many roles, the HIV-1 accessory protein Vpu performs a viroporin function and also antagonizes the host cell restriction factor tetherin through its transmembrane domain. BIT225 is a small molecule inhibitor that specifically targets the Vpu viroporin function, which, in macrophages, resulted in late stage inhibition of virus release and decreased infectivity of released virus, a phenotype similar to tetherin-mediated restriction. Here, we investigated whether BIT225 might mediate its antiviral function, at least in part, *via* inhibition of Vpu-mediated tetherin antagonism. Using T-cell lines inducible for tetherin expression, we found that BIT225 does not exert its antiviral function by inhibiting Vpu-mediated tetherin downmodulation from the cell surface, the main site of action of tetherin activity. In addition, results from a bioluminescence resonance energy transfer (BRET) assay showed that the Vpu-tetherin interaction was not affected by BIT225. Our data provide support for the concept that tetherin antagonism and viroporin function are separable on the Vpu transmembrane and that viroporin function might be cell-type dependent. Further, this work contributes to the characterization of BIT225 as an inhibitor that specifically targets the viroporin function of Vpu.

## Introduction

The human immunodeficiency virus 1 (HIV-1) has a complex retroviral genome, which, in addition to encoding the classical structural and enzymatic proteins Gag, Gag-Pol, Pol and Env, and the regulatory proteins Tat and Rev, also encodes the four accessory proteins Vpr, Vif, Vpu and Nef that play multiple roles in HIV-1 pathogenesis (reviewed in [1], [2]). An important function of the HIV-1 accessory proteins appears to be the antagonism of host cell restriction factors [3], [4], [5], [6], [7], [8], [9].

The viral protein Vpu is a 16 kDa type I transmembrane protein, consisting of a N-terminal transmembrane domain (AA 1–27) and a cytoplasmic domain (AA 28–81) of two consecutive amphiphatic α-helices (AA 33–49 and AA 57–70) [2], [10], [11]. At the cell membrane, Vpu assembles to a multimeric state, most likely as pentamers, but possibly also as tetramers or hexamers [11], [12], [13]. The most studied function of Vpu is the downmodulation of CD4, which permits Env trafficking to the viral assembly site and subsequent incorporation into the viral membrane. This CD4 downmodulation occurs in the endoplasmic reticulum (ER) and is mediated by the C-terminal domain of Vpu acting as a transient adaptor protein to link CD4 to β-transducin repeats-containing protein (β-TrCP), resulting in proteasomal degradation of CD4 but not of Vpu (reviewed in [14]).

A second function of Vpu is the antagonism of the host cell restriction factor tetherin (BST-2/CD317/HM1.24). Tetherin inhibits viral replication late in the viral replication cycle, inhibiting the budding of nascent virus by directly holding the budding virus to the cell surface [15], [16], [17]. Tetherin is constitutively expressed in various cells, including monocyte-derived macrophages, activated CD4^+^ T-cells and T-cell lines [18], [19], [20], [21], [22], [23]. Both this tetherin-mediated restriction as well as tetherin cell surface expression are interferon responsive, linking tetherin to the innate immune response [5], [15], [16], [23].

Tetherin is a 30–36 kDa type II transmembrane protein that consists of a short cytoplasmic N-terminal region (AA 1–21), a transmembrane region (AA 22–43), an ectodomain (AA 44–160), and a C-terminal glycosylphosphatidylinostol (GPI) anchor [19], [24]. Tetherin localizes to the plasma membrane, the trans-Golgi network (TGN) and the early and recycling endosomes, and cycles between these membrane compartments [24], [25]. Tetherin-mediated restrictive activity has commonly been attributed to its cell surface expression, though additional surface-independent mechanisms have been suggested but not yet characterized [5], [15], [21], [23], [26], [27].

In HIV-1 infection, the viral protein Vpu antagonizes tetherin-mediated restriction and promotes down-modulation of tetherin from the cell surface where viruses assemble and bud [28], [29]. Vpu-mediated downmodulation of tetherin can occur via tetherin degradation by the proteasome and/or the lysosome, and the sequestration of tetherin in intracellular compartments. For the degradation of tetherin, Vpu employs β-TrCP that acts in a fashion similar to that which occurs during degradation of CD4. Vpu recognizes tetherin through an interaction between the transmembrane domains of these two proteins. Molecular mapping revealed a few amino acids on each transmembrane domain that are crucial for functional interactions (Vpu: A14, A18 and W22) [30], [31], [32], [33], [34], [35], [36]. In Vpu transmembrane multimers, these residues are predicted to be outside-facing [33]; modeling of the tetherin transmembrane domain indicates a sided positioning of crucial amino acid residues in the helix and supports the existence of a direct Vpu-tetherin interface [5].

In addition to tetherin antagonism and virus release, the transmembrane domain of Vpu also functions as a cation-selective ion channel (also called viroporin) in a multimeric state [11], [37], [38], [39], [40]. Interestingly, the A18H mutation of an outside-facing residue important for Vpu-tetherin interaction rendered the viroporin activity of Vpu sensitive to rimantadine, an inhibitor of the viroporin function of influenza A M2 protein [41]; this suggests a possible link between Vpu viroporin function and Vpu-mediated promotion of virus release by tetherin antagonism. However, a recent study reported that tetherin antagonism and viroporin function are separable functions of Vpu. Mutation of the Vpu amino acids A14 and A18 to asparagines abrogated tetherin antagonism without affecting viroporin function [42]. Also, an S23A mutation eliminated viroporin function but did not affect anti-tetherin activity [42], [43].

BIT225 (N-[5-(1-methyl-1H-pyrazol-4-yl)-napthalene-2-carbonyl]-guanidine: CASNo. 917909-71-8) is a novel small molecule inhibitor of HIV-1 Vpu viroporin function. In addition to its activity against Vpu, BIT225 also abrogates the viroporin function of hepatitis C virus (HCV) protein p7 [44], [45]. Further, BIT225 displays a synergistic effect in HCV infections with interferon α2b (IFNα2b) *in vitro* [44], which also stimulates tetherin expression as part of the interferon-induced antiviral state [23], [32]. In monocyte-derived macrophages (MDMs), which express high levels of endogenous tetherin and which represent a long-lived virus producing reservoir in HIV-1 infection, BIT225 efficiently blocks HIV-1 virus release and reduces the infectivity of released virus [45], [46]. Tetherin also inhibits the release of Δ*vpu* virus and renders released virus less infectious [15], [16], [32]. Interestingly, BIT225 exerts higher antiviral efficacy in MDMs than in CD4^+^ T-cells, even though the latter express lower endogenous tetherin levels [45].

Therefore, we have now investigated whether the antiviral activity of BIT225 might be partly related to inhibition of Vpu-mediated tetherin-antagonism in tetherin expressing CD4^+^ T-cell lines. However, we were not able to detect a tetherin-mediated impact on BIT225 function, which suggests that BIT225 specifically blocks Vpu viroporin function. These data also support the concept that viroporin function and virus release are separable functions of the Vpu transmembrane domain [42], and that the viroporin function of Vpu may be cell type specific [45].

## Results

**Figure 1:**
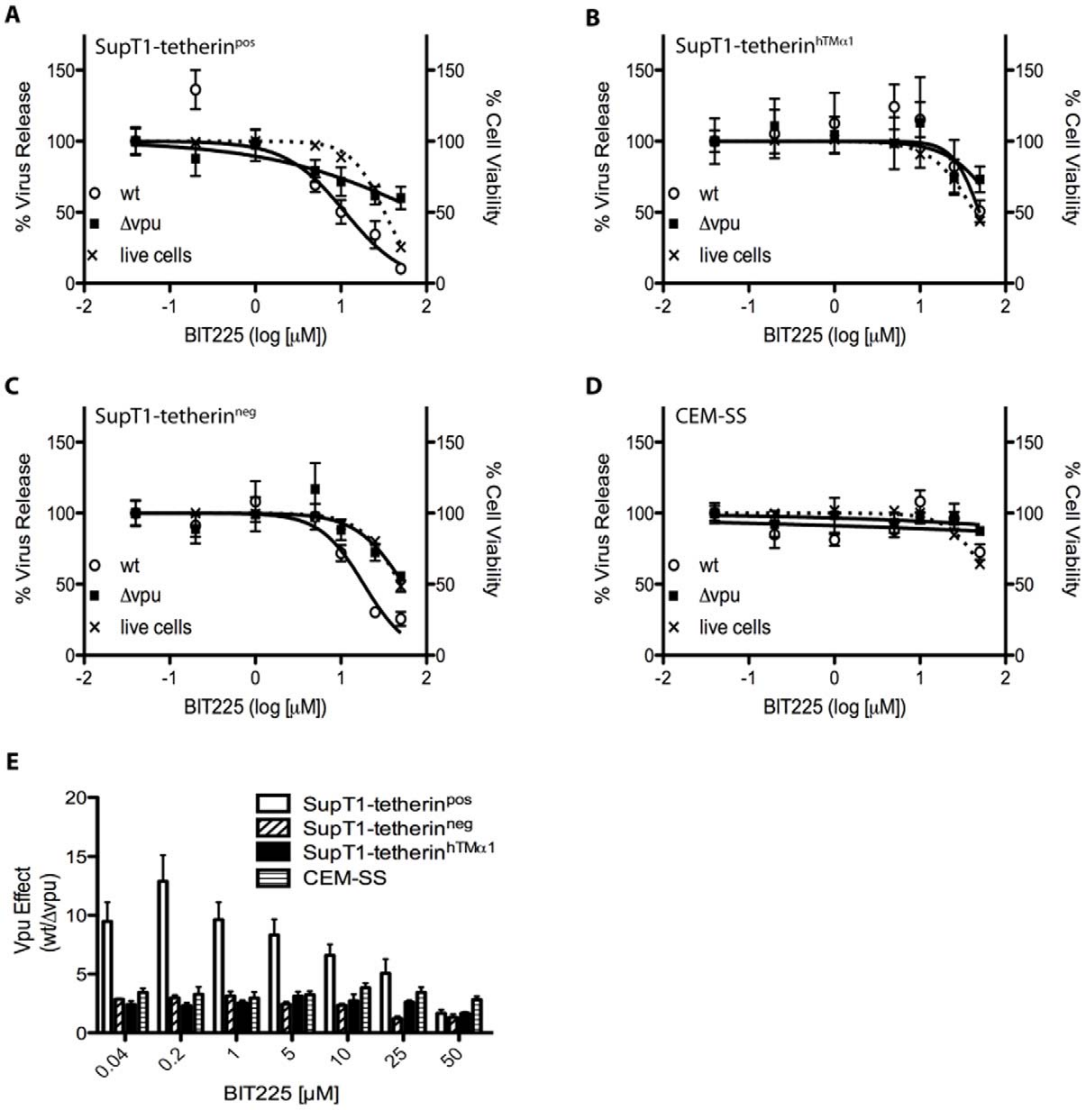
BIT225 does not affect Vpu-mediated antagonism of tetherin-mediated restriction of virus release independent of cell toxicity. The T-cell lines SupT1-tetherin^pos^ (**A**), SupT1-tetherin^hTMα1^ (**B**), SupT1-tetherin^neg^ (**C**) and CEM-SS cells (**D**) were infected with equal amounts of *wt* or Δ*vpu* virus by spinoculation and cultured in media containing BIT225 at concentrations of 0.04, 0.2, 1, 5, 10, 25 and 50 µM. Virus release was assessed at 72 h p.i. by reverse transcriptase assay (solid lines). Cell viability was assessed by flow cytometry using side scatter and forward scatter in the presence of BIT225 (0.04, 0.2, 1, 5, 10, 25 and 50 µM) compared to DMSO controls (dotted lines) (**A-D**). Normalized data from three independent experiments, analyzed for non-linear regression, are presented; error bars represent standard error of the mean (SEM). (**E**) The relative Vpu-mediated impact of virus release from A-D was determined by normalizing virus release in wt infected populations relative to virus release in Δ*vpu* infected populations.

First we assessed the cyototoxicity of BIT225 on the following T-cell lines: SupT1-tetherin^pos^ (transduced with human tetherin), SupT1-tetherin^neg^ (transduced with an empty vector), SupT1-tetherin^hTMα1^ (transduced with a chimeric tetherin resistant to Vpu mediated antagonism), and CEM-SS cells [23], [32], [47]. CEM-SS cells express detectable endogenous levels of cell surface tetherin, and this correlates with their lower permissiveness to Δ*vpu* viral replication compared to *wt* viral replication [23]. Cells were cultured in media containing BIT225 at concentrations of 0.04, 0.2, 1, 5, 10, 25 or 50 µM for 72 h, after which we assessed cell viability using flow cytometry detection of side scatter (SSC) and forward scatter (FSC), comparing BIT225 treated populations to dimethyl sulfoxide (DMSO) treated controls (Figure S1). Non-linear regression analysis revealed that BIT225 had a 50% cytotoxic concentration of 32–50 µM in the transduced Sup-T1 cell lines (Fig. 1A–C, Table 1). CEM-SS cells showed reduced sensitivity to BIT225 (extrapolated 50% cytotoxic concentration: 67 µM) (Fig. 1D; Table 1).

**Table 1:** IC50 concentrations and 50% viability concentrations of BIT225 determined by non-linear regression analysis.

T-cell line	50% cell viability (BIT225 [µM])	IC50 – *wt* (BIT225 [µM])	IC50 - Δ*vpu* (BIT225 [µM])
**SupT1-tetherin^neg^**	49.48	18.44	54.19
**SupT1-tetherin^pos^**	32.25	11.15	89.97
**SupT1-tetherin^hTMα1^**	43.67	49.59	85.51
**CEM-SS**	67.21	>>100	>>100

We then assessed the inhibitory effect of BIT225 on virus release in these cell lines infected with equal amounts of *wt* or Δ*vpu* virus and cultured in media containing BIT225 at concentrations of 0.04, 0.2, 1, 5, 10, 25 or 50 µM at 72 h post infection (p.i.) (Fig. 1, Table 1). The half maximal inhibitory concentrations (IC50) for virus release in *wt* infected populations were ∼18 µM in SupT1-tetherin^neg^ cells, ∼11 µM in tetherin expressing SupT1-tetherin^pos^ cells and ∼50 µM in the SupT1-tetherin^hTMα1^ control cell line, expressing chimeric tetherin, resistant to Vpu antagonism. Regarding Δ*vpu* infected populations, the extrapolated IC50 concentrations were ∼54 µM (SupT1-tetherin^neg^), ∼90 µM (SupT1-tetherin^pos^) and ∼86 µM (SupT1-tetherin^hTMα1^), respectively (Fig. 1A–C; Table 1). To assess the impact of BIT225 on Vpu-mediated virus release (‘Vpu effect’), we normalized virus release in *wt* infected populations to that of Δ*vpu* infected populations (Fig. 1E). In SupT1-tetherin^neg^ cells, the ‘Vpu effect’ remained stable at 2.4–3 at BIT225 concentrations up to 10 µM, after which it declined to ∼1.2, which indicates almost equal virus release in *wt* and Δ*vpu* infected populations. A similar trend was observed in the Vpu-resistant SupT1-tetherin^hTMα1^ cells, expressing tetherin, though the reduction only occurred at 25 µM (fold change: ∼1.6). In CEM-SS cells, virus release in *wt* and Δ*vpu* infected populations was not significantly affected by BIT225 concentrations of up to 50 µM, and the ‘Vpu effect’ remained relatively stable at ∼3–3.5 fold (Fig. 1D; Table 1).

In SupT1-tetherin^pos^ cells, however, the Vpu effect was generally stronger at concentrations up to 5 µM (0.04 µM: 9.2, 5 µM: 8.5), with a peak at 0.2 µM BIT225 (ratio: 12). Starting at 10 µM the ratio decreased (10 µM: 6.5, 25 µM: 5, 50 µM 1.6) to levels similar to those obtained with the other cell lines (Fig. 1E).

**Figure 2:**
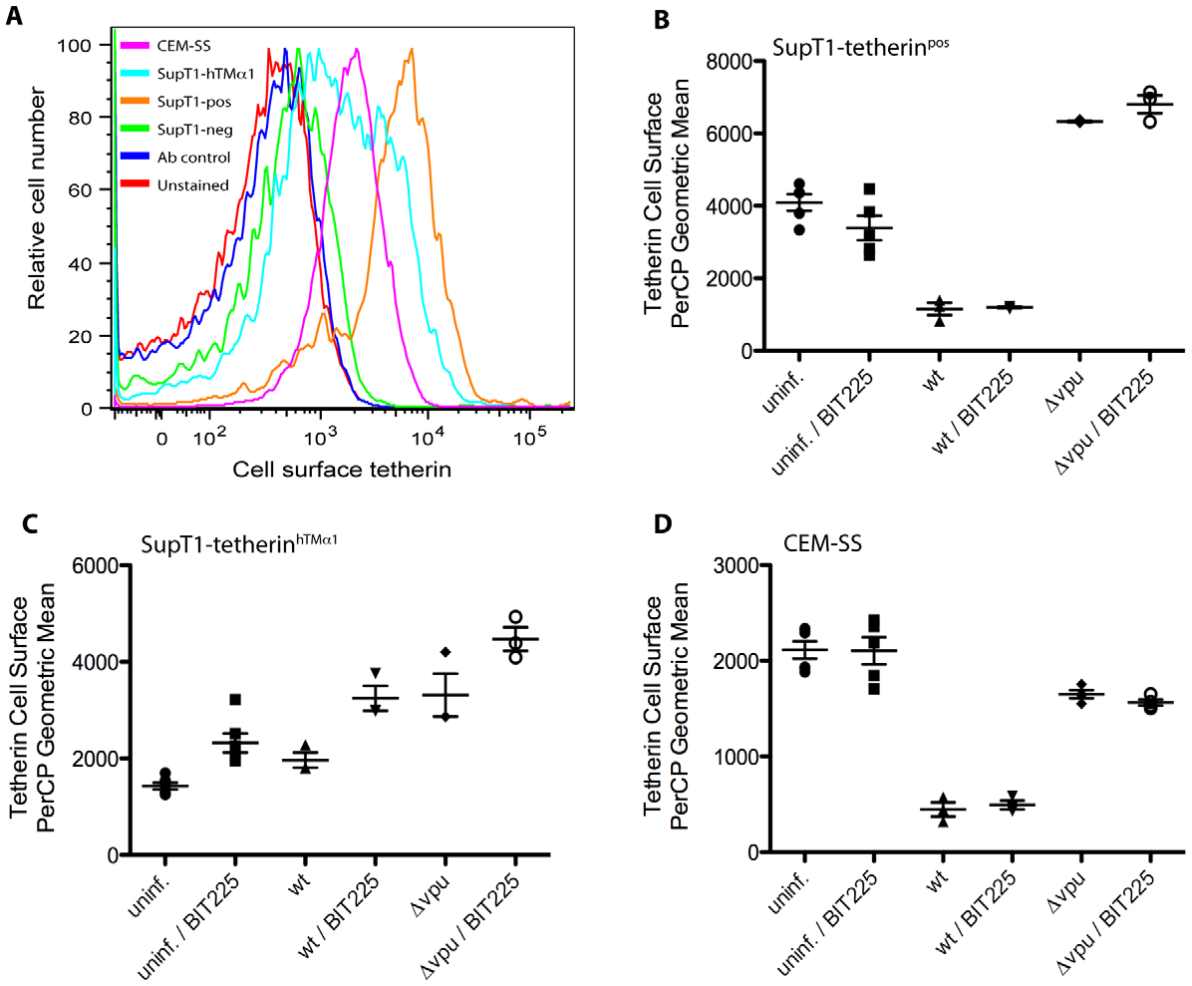
BIT225 does not modulate tetherin cell surface expression. (**A**) Representative overlay of tetherin cell surface expression levels. Cell surface tetherin levels assessed by flow cytometry detection of PerCP-levels in SupT1-tetherin^pos^ cells (*orange*), SupT1-tetherin^hTMα1^ cells (*turquoise*), SupT1-tetherin^neg^ (green) and CEM-SS cells (*rose*). Controls are unstained SupT1-tetherin^pos^ cells (*red*) and SupT1-tetherin^pos^ cells stained with secondary antibody only (*blue*). (**B–D**) Geometric means of cell surface expression of tetherin in SupT1-tetherin^pos^ cells (**B**), SupT1-tetherin^hTMα1^ cells (**C**) and CEM-SS cells (**D**) in the presence of BIT225 [10 µM] or absence of BIT225 (DMSO control). Cells were infected with equal amounts of *wt* and Δ*vpu* BR-NL43-IRES-eGFP. At 48 h p.i., cells were gated into uninfected and infected populations, based on their virus-derived eGFP expression profile, and cell surface levels of tetherin were assessed. Data are derived from a minimum of three independent experiments.

The apparently elevated sensitivity of SupT1-tetherin^pos^ cells to BIT225 might be due to an inhibition of Vpu-mediated tetherin antagonism. Therefore, we assessed whether BIT225 might influence tetherin cell surface expression levels in uninfected cells and/or might affect tetherin modulation following infection. Cells were infected with *wt* or Δ*vpu* virus and cultured in the absence (DMSO control) or presence of 10 µM BIT225, a concentration almost equal to the IC50 in SupT1-tetherin^pos^ cells for virus release. At 72 h p.i., tetherin cell surface expression in infected and uninfected cells was assessed using flow cytometry; infected and uninfected cells were distinguished based on virus-derived enhanced green fluorescent protein (eGFP) expression (Figure S2). We were able to specifically detect and quantify tetherin cell surface expression and its modulation following infection in SupT1-tetherin^pos^ cells, SupT1-tetherin^hTMα1^ cells, and CEM-SS cells (Fig. 2, Figure S2); cell surface tetherin expression in SupT1-tetherin^neg^ cells was at the limit of specificity of detection (Fig. 2A). In *wt* infected SupT1-tetherin^pos^ cells, tetherin surface expression was downregulated by ∼70% compared to uninfected controls. In Δ*vpu* infected cells, detection of cell surface tetherin was increased (∼50%), as previously reported [47]. Treatment with 10 µM BIT225 did not affect tetherin cell surface levels in uninfected cells, neither did BIT225 affect either Vpu-mediated tetherin downmodulation in *wt* infection or upregulation in Δ*vpu* infected SupT1-tethern^pos^ cells. In SupT1-tetherin^hTMα1^ cells, tetherin levels were not downregulated following *wt* infection compared to uninfected cells, but slightly upregulated (∼35%); infection with Δ*vpu* virus resulted in an upregulation of cell surface tetherin by ∼120%. Cell surface tetherin levels followed the same trend in BIT225 treated cells, but were generally increased, compared to the respective untreated populations (uninfected: 65% increase; *wt* infections: 60% increase; Δ*vpu* infections: 35% increase). In CEM-SS cells, *wt* infection decreased cell surface tetherin by ∼75%, while Δ*vpu* infected cells showed a decrease of ∼20% in cell surface tetherin expression, compared to uninfected populations, as previously described [23]. BIT225 did not affect tetherin cell surface levels in uninfected CEM-SS cells or tetherin modulation in infected cells.

**Figure 3:**
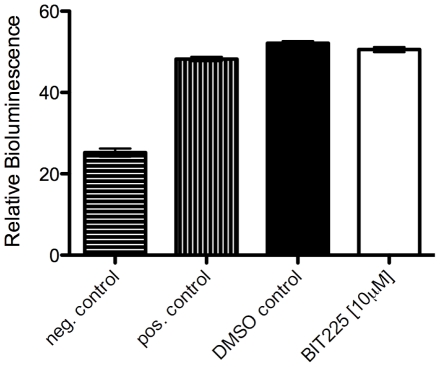
BIT225 does not affect Vpu-tetherin interactions. A bioluminescence resonance energy transfer (BRET) assay was used to study Vpu-tetherin interaction. Tetherin fused to RLuc (*Renilla* luciferase) and Vpu fused to eYFP (enhanced yellow fluorescent protein) were transfected into 293T cells. At 48 h post transfection, eYFP emission was detected following RLuc excitation, representing energy transfer between these two proteins at close proximity (<10 Å). BRET assays were performed in the presence of BIT225 [10 µM] or its absence (DMSO control). The negative control represents cotransfection of an empty (Vpu-negative) eYFP vector and the tetherin-RLuc vector; the positive control is a vector coding for eYFP-RLuc-fusion protein. Data are derived from a minimum of three independent experiments, performed in duplicate; error bars represent SEM.

To further test whether BIT225 might affect Vpu-tetherin interactions, we performed a bioluminescence resonance energy transfer assay (BRET). In this assay, energy transfer is observed only upon close proximity (<10 Å) of *Renilla* luciferase (RLuc)-fused tetherin and enhanced yellow fluorescent protein (eYFP)-fused Vpu, indicating a direct interaction. A disruption of interaction results in a decreased eYFP emission signal following luciferase excitation. The assay was performed in the absence (DMSO control) or in the presence of 10 µM BIT225, which is close to the IC50 in regard to virus release in tetherin expressing SupT1-tetherin^pos^ cells. We were able to specifically detect bioluminescence energy transfer in our system. The negative control, wherein an eYFP protein is used in the absence of Vpu, resulted in a background signal of ∼25; the positive control, provided by a RLuc-eYFP fusion protein, exhibited a relative signal of ∼49. The use of RLuc-fused tetherin and eYFP-fused Vpu resulted in a signal of ∼52 in the absence of BIT225 and a signal of ∼51 in the presence of BIT225 (Fig. 3). Although these ratios were not significantly different from each other nor different from the positive control, the difference from the negative control was significant. Thus, while the BRET assay indicates a specific Vpu-tetherin interaction, BIT225 does not appear to influence Vpu-tetherin interactions in this system, confirming the absence of Vpu-tetherin modulation by BIT225 in regard to virus release and tetherin cell surface expression.

## Discussion

BIT225 is a novel antiviral compound that inhibits the viroporin function of HIV-1 Vpu and HCV p7 [44], [45]. In HIV-1 infections, MDMs represent an important active viral reservoir [48]. Treatment of MDMs with BIT225, and subsequent inhibition of Vpu (viroporin function), inhibits viral replication at a late stage and reduces virus release and viral infectivity [45], [46]. HIV-1 infection of T-cells proved to be less sensitive to BIT225-mediated inhibition of Vpu [45]. In HCV infections, BIT225 is synergistic with IFNα2b *in vitro*, which also induces tetherin expression across a wide range of cells including CD4^+^ T-cells, the primary target for HIV-1 [16], [21], [23], [32], [44]. Several CD4^+^ T-cell lines, including CEM-SS, display detectable levels of endogenous tetherin at the cell surface, which correlates with their reduced permissiveness towards Δ*vpu* replication [23]. Tetherin is a host cell restriction factor that acts late in the viral life cycle, inhibiting release of nascent virus by directly linking the viral and cellular membranes [15], [16], [26]. In HIV-1 infection, Vpu antagonizes tetherin-mediated restriction by downmodulating tetherin from the cell surface [15], [26]; compared to CD4^+^ T-cells, MDMs express higher endogenous tetherin levels than CD4^+^ T-cells [21], [23]. Tetherin also reduces the infectivity of released virus and there is evidence that tetherin also inhibits direct cell-to-cell spread of HIV-1 [47], [49], though the extent of this latter effect is still debated [50].

Vpu mediates its viroporin function and its tetherin antagonizing activity via its transmembrane domain. Although the Vpu transmembrane domain is responsible for both the viroporin function and tetherin antagonism, a recent report suggests that these two activities can be distinguished [42]. Here, we investigated whether the Vpu-specific inhibitor BIT225 might partially act *via* inhibition of Vpu-mediated tetherin antagonism, in addition to its known inhibitory effect on Vpu viroporin function [38], [45], [46]. To this end, we used a panel of SupT1 T-cell lines which are inducible for expression of human tetherin (SupT1-tetherin^pos^) or a Vpu-resistant tetherin variant, resistant due to a chimeric transmembrane domain (SupT1-tetherin^hTMα1^); the control cell line was transduced with an empty vector and tetherin levels in that cell line were below the sensitivity level of tetherin detection by flow cytometry [32], [47]. In this panel, tetherin expression is independent of the multifaceted IFN response. Therefore, this system allows the specific investigation of tetherin-mediated effects and Vpu-mediated countermeasures. In addition, a CEM-SS cell line, which expresses intermediate levels of tetherin was used [23]. The effects of BIT225 on cell viability, virus release, Vpu-tetherin interaction and Vpu-mediated downmodulation from the cell surface were investigated.

BIT225 exerts similar effects on the viability of SupT1-tetherin^neg^ cells and SupT1-tetherin^hTMα1^ cells, while the tetherin expressing SupT1tetherin^pos^ cell line was more sensitive to BIT225, exhibiting reduced viability at lower concentrations. As the only difference between these cell lines is the expression of tetherin, the reduced cell viability of these SupT1-tetherin^pos^ cells in response to BIT225 may be due to higher tetherin expression in these cells, leading to their increased fragility when cultured with drug (Fig. 2A). Also, all SupT1 cell lines showed decreased viability in the presence of BIT225 when compared to CEM-SS cells (Fig. 1; Table 1). Transduced SupT1-based cell lines need to be cultured in the presence of puromycin, G418 and doxycycline to maintain the inserted tetherin gene and to induce tetherin expression. It is not known whether these compounds might sensitize cells to BIT225 or whether drug interactions might occur in this circumstance.

In the SupT1 cell lines examined, BIT225 inhibited virus release in a Vpu-specific manner; BIT225 IC50 concentrations were lower in the presence of Vpu, comparing *wt* to Δ*vpu* infections (Fig. 1; Table 1). Since the IC50 values were less than 3-fold lower than the extrapolated 50% cytotoxic concentrations of BIT225 in the Sup-T1 cell lines, and to achieve a more meaningful readout, we also calculated the relative impact of Vpu (‘Vpu effect’) by normalizing virus release in *wt-*infected compared to Δ*vpu-*infected populations over a range of BIT225 concentrations. At low concentrations of BIT225, Vpu promoted virus release in all SupT1 cell lines, an effect that was antagonized by increasing the concentration of BIT225 (Fig. 1E). As the SupT1-tetherin^neg^ cell line does not exhibit detectable levels of tetherin expression (Fig. 2A), the Vpu-mediated impact is due to the viroporin function of Vpu. Therefore, it is reasonable to argue that the decreased impact of Vpu on virus release in the presence of increasing levels of BIT225 is due to BIT225-mediated inhibition of Vpu viroporin function. The same argument applies to the SupT1-tetherin^hTMα1^ cell line, transduced with a tetherin variant that is resistant to Vpu-mediated antagonism.

In this assay system and without the addition of drug, the increased Vpu effect on virus release in SupT1-tetherin^pos^ cells compared to the other cell lines, is due to the Vpu-mediated tetherin antagonism, which promotes virus release [15], [16], [23], [32]. The Vpu effect appeared to be more sensitive to BIT225 in SupT1-tetherin^pos^ cells compared to the other SupT1 cell lines. However, the similarity of the BIT225 therapeutic ratios (50% viability concentration/IC50 concentration) in *wt* infection of SupT1-tetherin^pos^ (2.89) and SupT1-tetherin^neg^ (2.68) cells does not support such an interpretation.

In the CD4^+^ T-cell line CEM-SS, Vpu antagonized tetherin-meditated restriction of virus release and downmodulated tetherin from the cell surface (Fig. 1E & Fig. 2A&D [23]). Interestingly, BIT225 did not cause detectable toxicity in this cell line. Also, and in contrast to the panel of Sup-T1 cell lines, the ‘Vpu effect’ in CEM-SS remained stable over the range of BIT225 concentrations used (Fig. 1E & Fig. 2A&D). Taken together, this suggests a reduced need for Vpu viroporin function in CEM-SS cells for viral replication, and might also reflect a potential alteration of a cellular function in this immortalized cell line, rendering it less sensitive to BIT225 toxicity. The dependence of virus release on Vpu-mediated tetherin antagonism, paired with apparent insensitivity towards inhibition of Vpu-viroporin function in CEM-SS cells, further supports the concept of tetherin antagonism and viroporin activity as being separable functions of Vpu.

Investigation of the Vpu-tetherin interrelationship by detection of Vpu-mediated tetherin cell surface downregulation was performed by flow cytometry (Fig. 2). Vpu-tetherin interactions were also studied using a BRET assay (Fig. 3). Both methods confirmed the absence of additional effects of BIT225 on Vpu-tetherin interactions. We have been able to induce tetherin expression in SupT1-tetherin^pos^ cells and SupT1-tetherin^hTMα1^ and to specifically detect cell surface tetherin expression using flow cytometry (Fig. 2A). In SupT1-tetherin^pos^ cells, *wt* infection led to tetherin cell surface downregulation, while cell surface tetherin was upregulated in cell populations following Δ*vpu* infection compared to uninfected cells, as previously described [47], [51]. The presence of BIT225 at levels of 10 µM neither affected cell surface tetherin expression in uninfected cells nor Vpu-mediated tetherin downmodulation or upregulation following Δ*vpu* infections (Fig. 2B). This shows that the sensitivity of *wt* infection (and the normalized ‘Vpu effect’) in regard to the tetherin expressing SupT1-tetherin^pos^ cell line is independent of interference with the Vpu function in tetherin antagonism. Interestingly, BIT225 mediated an increase in tetherin cell surface detection in SupT1-tetherin^hTMα1^ cells (Fig. 2C). This was true in both uninfected cells and cells infected with *wt* or Δ*vpu* virus, although the effect was less pronounced in the Δ*vpu* infected population. HIV-1 Vpu was not able to antagonize and downmodulate the hTMα1-tetherin variant from the surface of *wt* infected cells; rather cell surface levels increased, although to lower levels than in the Δ*vpu* infected cells. The resistance to Vpu-mediated downregulation is in agreement with Vpu-resistance and virus release (Fig. 1; [32]). This different tetherin expression profile in CEM-SS cells (Vpu-independent tetherin downmodulation) has been previously described and supports the existence of additional anti-tetherin mechanisms following infection, which appear to not be affected by BIT225 (Fig. 2D [21], [23]).

To examine the possible effect of BIT225 on Vpu-tetherin interactions, we employed a Vpu-tetherin BRET assay that specifically detects bioluminescence resonance energy transfer between Vpu and tetherin when both are in immediate proximity (<10 Å), indicative of direct interaction. Our findings support the concept that Vpu and tetherin interact directly (Fig. 3) [15], [16], [26], [30], [31], [32], [33]. However, BIT225 did not modulate the BRET signal, indicating that BIT225 does not interfere with Vpu-tetherin interaction (Fig. 3). As Vpu in regard to tetherin antagonism might be outside-facing in Vpu multimers whereas viroporin function is believed to be focused at the inner face of Vpu multimers, the absence of BIT225-mediated Vpu-tetherin interactions gives support to the idea that BIT225 specifically targets the viroporin function, most likely on the inside of Vpu multimers.

Furthermore, these results indicate that viroporin function and tetherin antagonism represent distinct functions of the Vpu transmembrane domain in agreement with a recent report that studied this topic using a mutagenesis approach [42]. These findings are also in agreement with the modeling of a putative Vpu-tetherin interaction surface, based on nuclear magnetic resonance (NMR) structures, and the discovery that amino acid residues necessary for tetherin antagonism are outside-facing on the Vpu multimer [5], [33], while residues crucial for viroporin function are predicted to face the inside of the Vpu multimer [42], [43]. However, both functions affect virus release and viral infectivity at a late stage in the viral replication cycle [32], [45].

Our data further support the characterization of BIT225 as a specific inhibitor of the viroporin function of Vpu and a potentially useful agent to target cellular viral reservoirs. Our results also imply that Vpu viroporin function may be cell type specific, as the efficacy of BIT225 has been reported to be greater in MDMs than in T-cells. However, specific work with MDMs may be difficult to achieve, due to a lack of functional MDM cell lines and a scarcity of knowledge on the role of tetherin in MDMs.

## Materials and Methods

### Cells

CEM-SS cells were obtained from the NIH AIDS Research and Reference Reagent Program [52] and were maintained in RPMI-1640 culture medium (Gibco) supplemented with 10% bovine serum albumin (BSA). Sup-T1 cells were also obtained from the NIH AIDS Research and Reference Reagent Program [52]. Transduced Sup-T1 cells were maintained in RPMI-1640 supplemented with 10% tetracycline-free BSA, 2 µg/ml puromycin (Sigma), and 1 mg/ml G418 (Sigma). Expression of human tetherin or its variant hTMα1 in Sup-T1 cells, was induced using 100 ng/ml doxycycline (Sigma) [32], [47], [51]; Sup-T1 cells stably transduced with the *wt* human tetherin gene (SupT1-tetherin^pos^), an empty vector (SupT1-tetherin^neg^), or the Vpu-resistant variant (SupT1-tetherin^hTMα1^), wherein the first nine amino acids of the transmembrane domain are replaced by the respective residues of tetherin from African green monkeys, were previously described [32].

### Viruses

Site-directed mutagenesis, using the QuickChange II XL Site-Directed Mutagenesis Kit (Stratagene), was used to introduce nucleotide changes into the coding regions of *vpu*, resulting in two stop codons at the beginning of Vpu coding regions of the viral clone pBR43-IRES-eGFP (NIH AIDS Research and Reference Reagent Program), expressing enhanced green fluorescent protein (eGFP) from an internal ribosomal entry site downstream of *nef* [53]. Virus was produced in 293T cells using Lipofectamine2000 (Invitrogen) as a transfection reagent. Virus was collected after 48 h, filtered (0.45 µm), and viral capsid/p24 protein (CA p24) content was quantified by a Vironostika HIV-1 Ag kit (bioMérieux).

### Compound

BIT225 (Batch 106R) was provided by Biotron Limited. The compound was dissolved in anhydrous dimethyl sulfoxide (DMSO) at 100 mM and was further diluted in culture media to working concentrations of 0.04, 0.2, 1, 5, 10, 25 or 50 µM.

### HIV-1 infections

Cell populations were infected with equal amounts of *wt* or Δ*vpu* virus to ∼10% infection rates, as determined by flow cytometric detection of virus-derived eGFP expression at 72 h p.i., in order to minimize superinfection events. Sup-T1 cells were infected with 600 ng CA p24 per 10^6^ cells (CEM-SS: 300 ng CA p24 per 10^6^ cells) by spinoculation (1,500 x g, at 37°C for 2 h), followed by incubation for 1 h at 37°C, after which virus was removed by centrifugation and cell washing. Cells (10^5^ cells/ml) were then cultured in media containing BIT225 at concentrations of 0.04, 0.2, 1, 5, 10, 25 or 50 µM. At 72 h p.i., virus release and tetherin cell surface expression were assessed.

### Virus release

Virus release into the supernatant was analyzed at 72 h p.i. using a quantitative reverse transcription-based assay [54]. Virus release was determined from populations infected with *wt* or Δ*vpu* virus, cultured in media containing BIT225 at concentrations of 0.04, 0.2, 1, 5, 10, 25 or 50 µM, or equivalent amounts of DMSO solvent only.

### Cell viability

Cell viability was determined through flow cytometry detection of forward scatter (FSC) and side scatter (SSC) of cells cultured in BIT225-containg media (0.04, 0.2, 1, 5, 10, 25 or 50 µM) or media with DMSO as a control for 72 h. Flow cytometry analysis was performed on a minimum of 30,000 cells using a LSR II instrument (Becton Dickinson) and FlowJo 7.5 software (Tree Star).

### Cell surface tetherin

Levels of cell surface tetherin expression in uninfected populations or populations infected with *wt* or Δ*vpu* virus were assessed by flow cytometry for peridinin chlorophyll protein (PerCP) at 72 h p.i.; populations were cultured either in the presence of 10 µM BIT225 or DMSO (0.1 µl/ml). Staining for cell surface tetherin was performed using a primary rabbit anti-human-tetherin polyclonal antibody (1∶3000) (NIH AIDS Research and Reference Reagent Program [21]), followed by a PerCP-labeled secondary goat anti-rabbit antibody (1∶250) (Santa Cruz Biotechnology). Cells were stained at 4°C for 30 min and fixed in 4% paraformaldehyde for 25 min. Uninfected and infected cells were distinguished by virus-derived eGFP expression. Flow cytometry analysis was performed on a minimum of 30,000 cells using a LSR II instrument (Becton Dickinson) and FlowJo 7.5 software (Tree Star).

### Vpu-tetherin bioluminescence resonance energy transfer assay (BRET)

Human tetherin was cloned into the pRLuc-C3 vector (BioSignal Packard). Vpu was cloned into the pEYFP-N1 vector (Clontech). The constructs were transfected into HEK293T cells using Lipofectamine2000 (Invitrogen) as a transfection reagent. After 6 h, transfection media were replaced by media containing either 10 µM BIT225 or DMSO (to equivalent amounts). Cells were collected 48 h post-transfection and washed twice in PBS. Transfer of bioluminescence was assessed using a Synergy™ 4 Multi-Mode Microplate Reader (Bioteck). To measure fluorescence of RLuc, coelenterazine H (Promega) was added to a final concentration of 5 µM in PBS. For measurement of EYFP only, cells were resuspended in PBS. The negative control used was an empty EYFP-N1 vector cotransfected with Rluc-tetherin and the positive control was a vector with EYFP fused to RLuc (EYFP-RLuc). A minimum of 10^5^ cells were analyzed from each experiment.

### Statistical analysis

Data from at least three independent experiments were analyzed utilizing GraphPad PRISM 5 software. Differences were analyzed for statistical significance using a one-way analysis of variance (ANOVA) with Bonferroni's post-test for groups and Student's t-test for pairs of data. GraphPad PRISM 5 software was also utilized to determine IC50 values in a non-linear regression analysis.

## Supporting Information

Figure S1 **Cell viability assessment.** SupT1-tetherin^pos^, SupT1-tetherin^hTMα1^, SupT1-tetherin^neg^ and CEM-SS cells were cultured in media containing BIT225 at concentrations of 0.04, 0.2, 1, 5, 10, 25 and 50 µM or DMSO. At 72 h p.i., cell viability was assessed, based on flow cytometry detection of forward scatter (FSC) and side scatter (SSC). Representative dot plots are shown.
(PDF)

Figure S2 **Gating strategy and readout for cell surface tetherin expression.** Representative gating for infected and uninfected cell populations and representative readout of cell surface tetherin modulation following infection are shown. SupT1-tetherin^pos^ cells, SupT1-tetherin^hTMα1^ cells, SupT1-tetherin^neg^ and CEM-SS cells were infected with equal amounts of *wt* BR-NL43-IRES-eGFP. At 48 h p.i., live cells were detected according to their flow cytometric FSC/SSC profiles. Live cells were gated into uninfected and infected populations, based on their virus-derived eGFP expression profile. Tetherin cell surface expression was determined in uninfected populations (*red*) and infected populations (*blue*) via detection of PerCP and presented as overlays. Geometric means of PerCP signal in uninfected and infected populations were assessed for relative quantification and comparison of cell surface expression levels of tetherin.
(PDF)
