# Supplementary figures and images for: The HIV-1 Vpu Viroporin Inhibitor BIT225 Does Not Affect Vpu-Mediated Tetherin Antagonism
Source: PLoS One. 2011 Nov 14;6(11):e27660. doi: 10.1371/journal.pone.0027660 (PMC3215742; doi:10.1371/journal.pone.0027660)

Figure S1

SupT1-tetherin<sup>pos</sup>

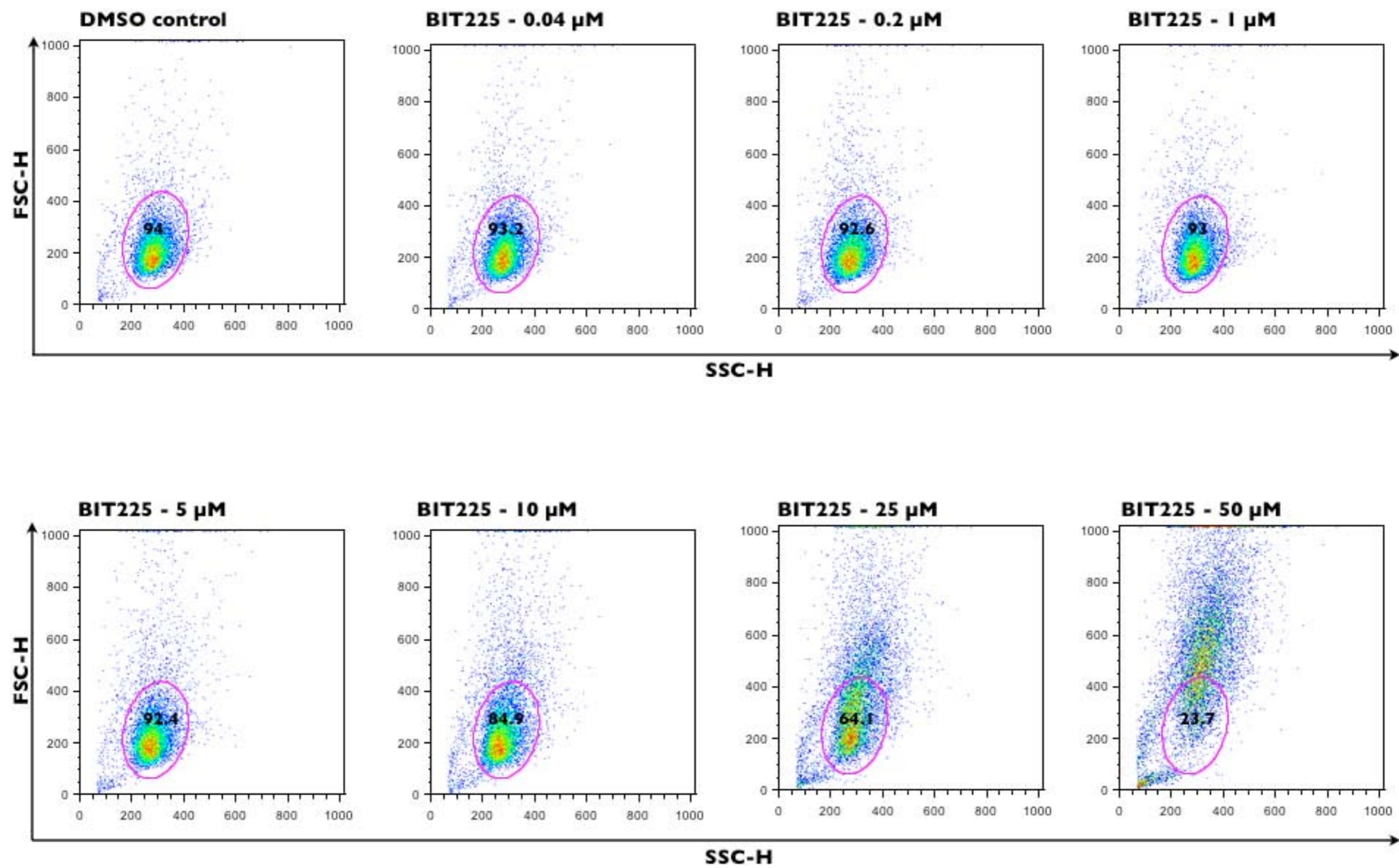

# SupT1-tetherin<sup>neg</sup>

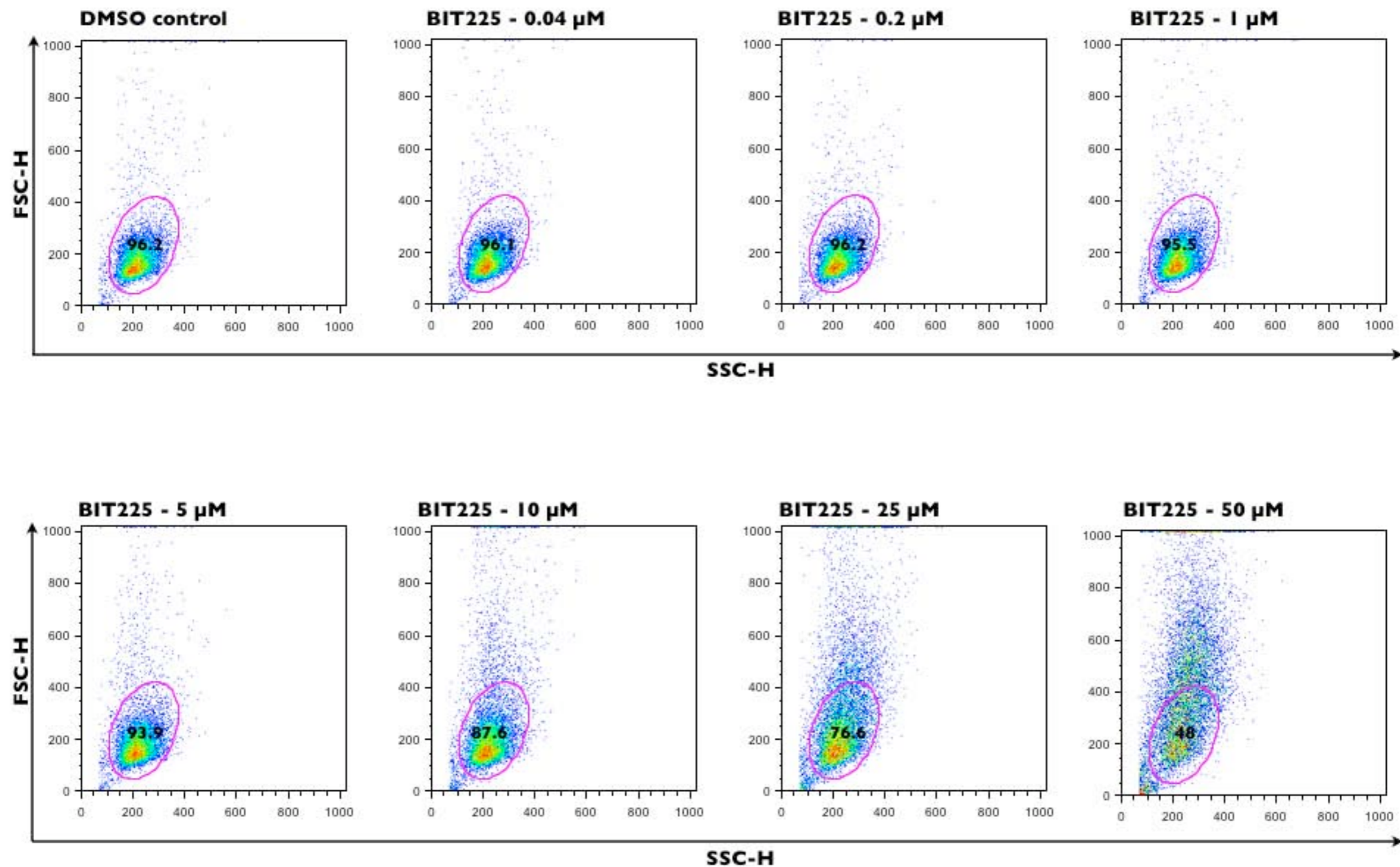

# SupT1-tetherin<sup>hTMA1</sup>

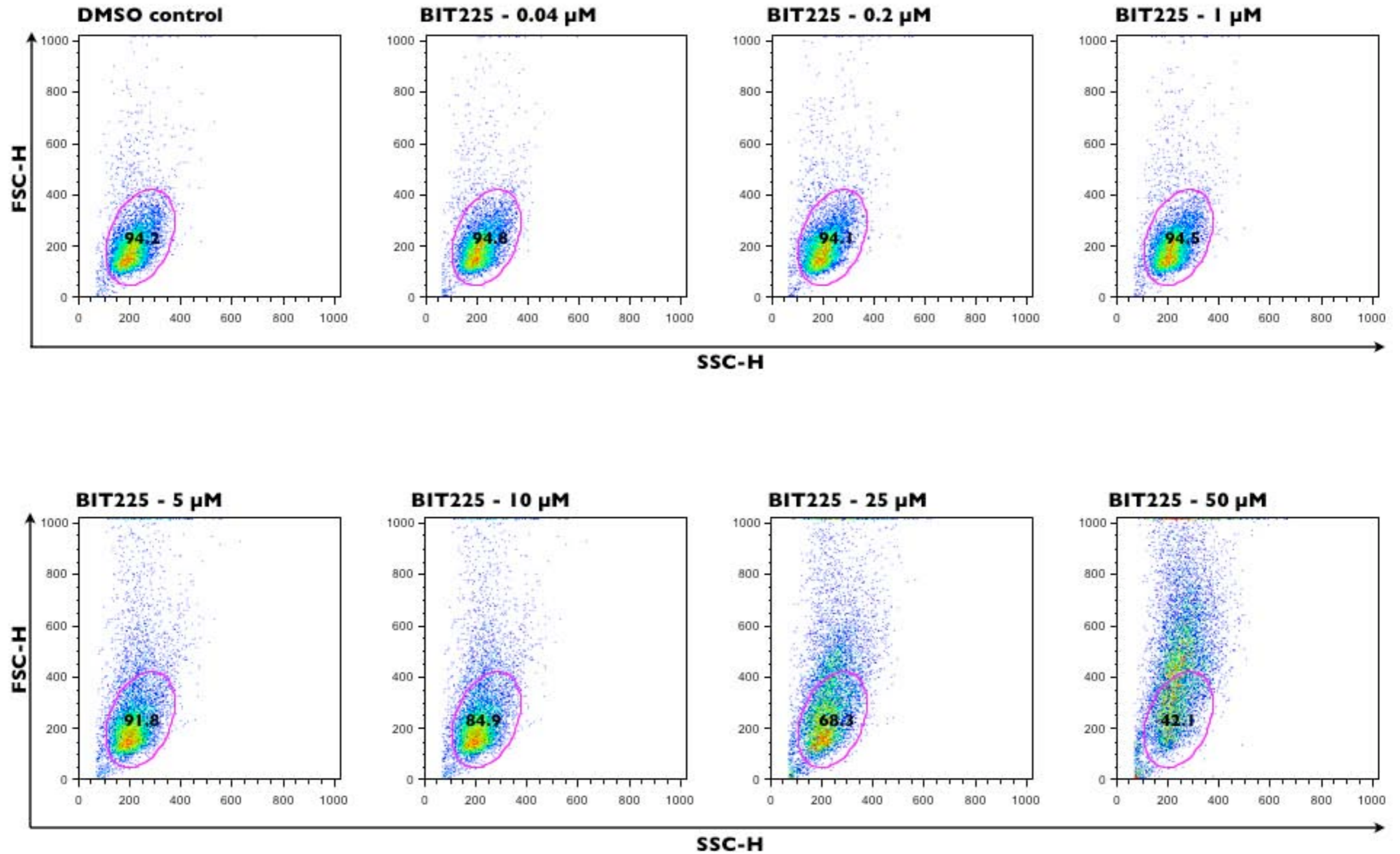

# CEM-SS

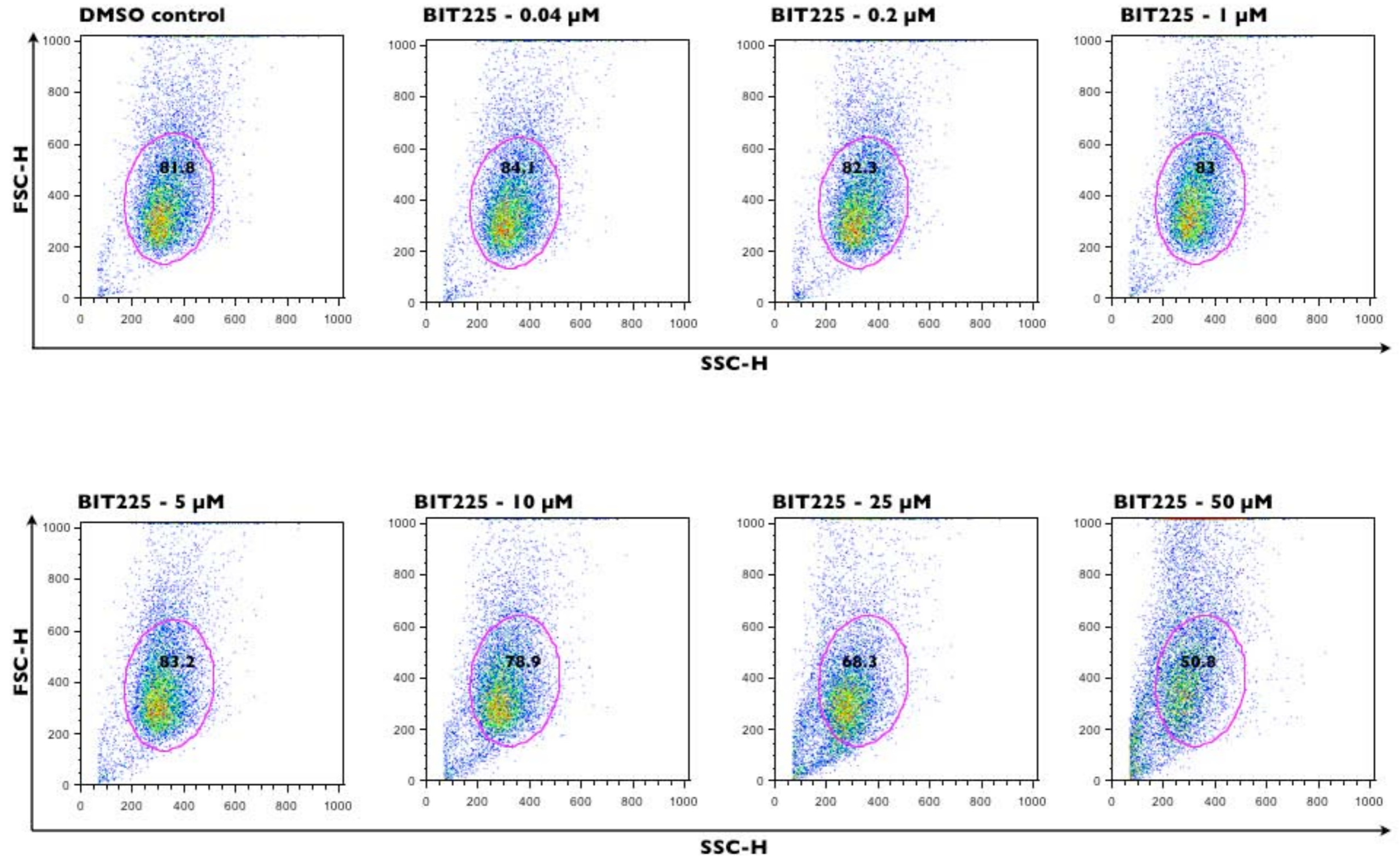

Supplement: Figure S1 — Cell viability assessment. SupT1-tetherinpos, SupT1-tetherinhTMα1, SupT1-tetherinneg and CEM-SS cells were cultured in media containing BIT225 at concentrations of 0.04, 0.2, 1, 5, 10, 25 and 50 µM or DMSO. At 72 h p.i., cell viability was assessed, based on flow cytometry detection of forward scatter (FSC) and side scatter (SSC). Representative dot plots are shown. (PDF) [file pone.0027660.s001.pdf]

Figure S2

SupT1-tetherin<sup>pos</sup>

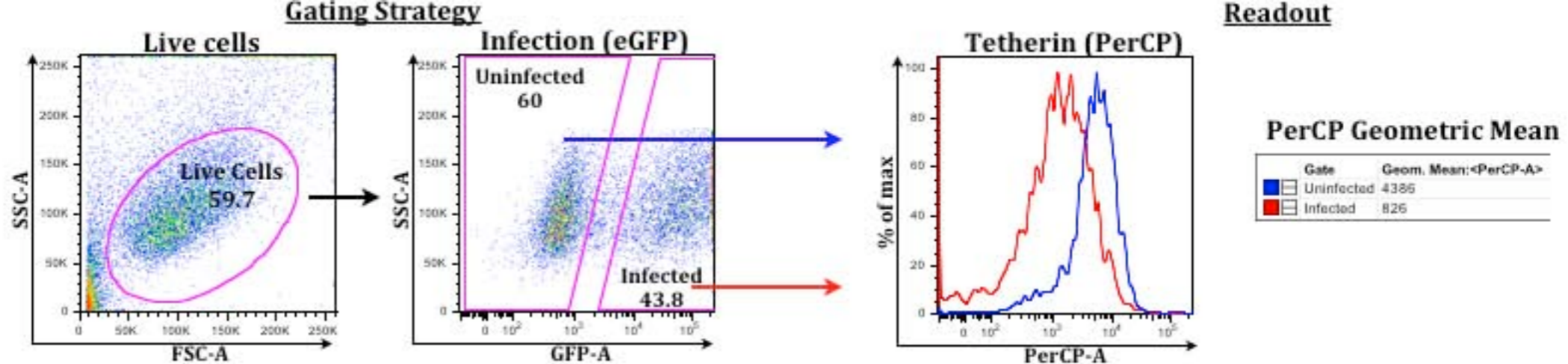

SupT1-tetherin<sup>neg</sup>

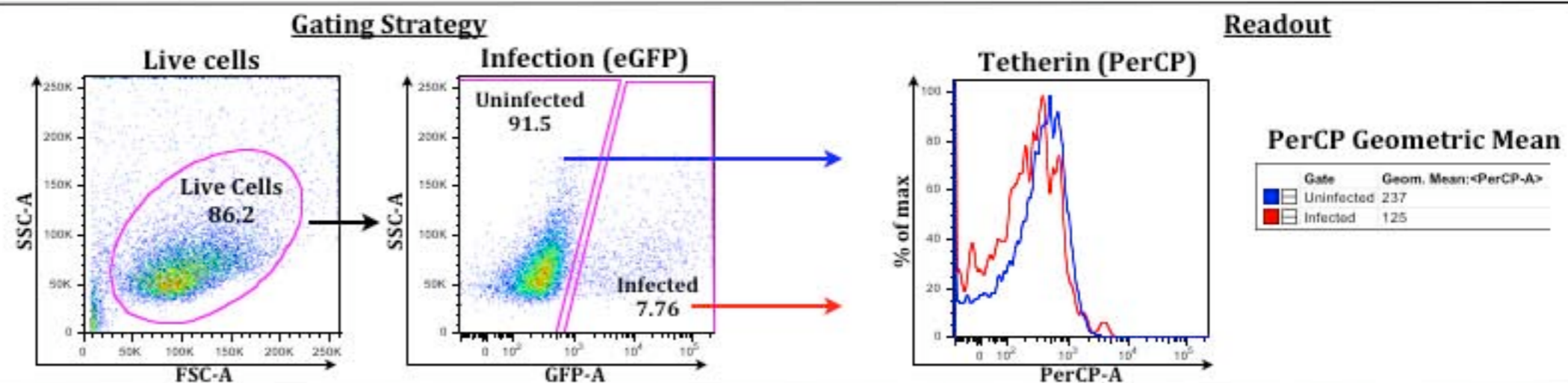

SupT1-tetherin<sup>hTMA1</sup>

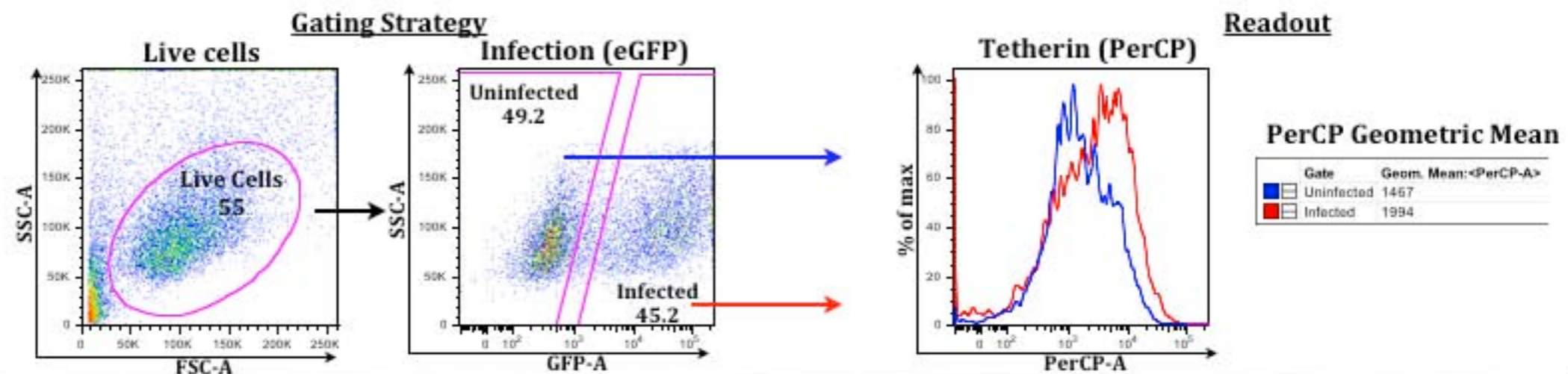

CEM-SS

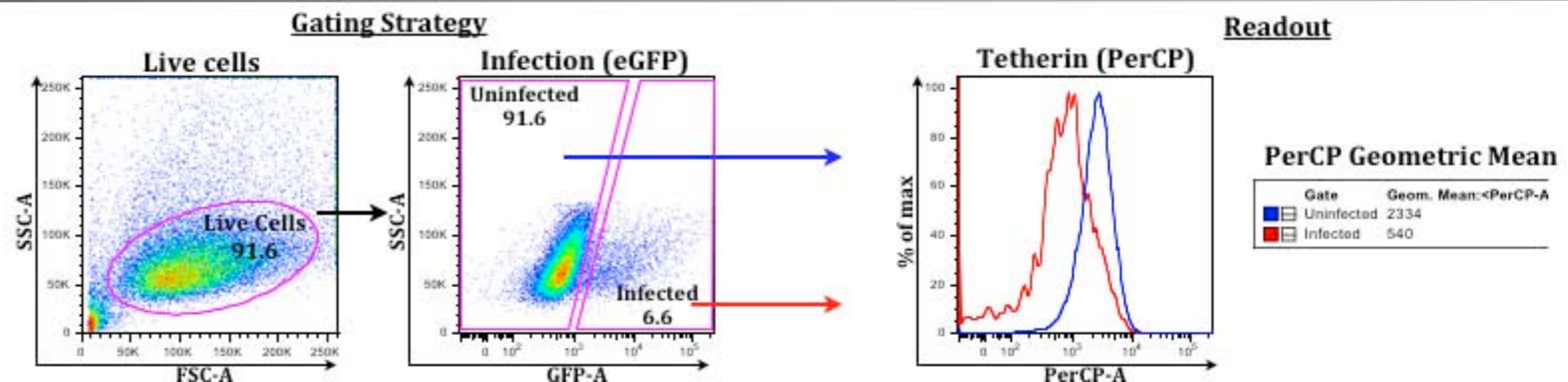

Supplement: Figure S2 — Gating strategy and readout for cell surface tetherin expression. Representative gating for infected and uninfected cell populations and representative readout of cell surface tetherin modulation following infection are shown. SupT1-tetherinpos cells, SupT1-tetherinhTMα1 cells, SupT1-tetherinneg and CEM-SS cells were infected with equal amounts of wt BR-NL43-IRES-eGFP. At 48 h p.i., live cells were detected according to their flow cytometric FSC/SSC profiles. Live cells were gated into uninfected and infected populations, based on their virus-derived eGFP expression profile. Tetherin cell surface expression was determined in uninfected populations (red) and infected populations (blue) via detection of PerCP and presented as overlays. Geometric means of PerCP signal in uninfected and infected populations were assessed for relative quantification and comparison of cell surface expression levels of tetherin. (PDF) [file pone.0027660.s002.pdf]
